# Detection of changes in gene regulatory patterns, elicited by perturbations of the Hsp90 molecular chaperone complex, by visualizing multiple experiments with an animation

**DOI:** 10.1186/1756-0381-4-15

**Published:** 2011-06-14

**Authors:** Pablo C Echeverría, Fedor Forafonov, Deo P Pandey, Guillaume Mühlebach, Didier Picard

**Affiliations:** 1 Département de Biologie Cellulaire, Université de Genève, Sciences 3, CH - 1211 Genève 4, Switzerland; 2 Biotech Research and Innovation Center (BRIC), University of Copenhagen, Ole Maaloes vej 5, 2200 Copenhagen, Denmark

**Keywords:** gene expression, microarray analysis, visualization, yeast, stress response, molecular chaperones, Hsp90, inhibitor, gene deletion

## Abstract

**Background:**

To make sense out of gene expression profiles, such analyses must be pushed beyond the mere listing of affected genes. For example, if a group of genes persistently display similar changes in expression levels under particular experimental conditions, and the proteins encoded by these genes interact and function in the same cellular compartments, this could be taken as very strong indicators for co-regulated protein complexes. One of the key requirements is having appropriate tools to detect such regulatory patterns.

**Results:**

We have analyzed the global adaptations in gene expression patterns in the budding yeast when the Hsp90 molecular chaperone complex is perturbed either pharmacologically or genetically. We integrated these results with publicly accessible expression, protein-protein interaction and intracellular localization data. But most importantly, all experimental conditions were simultaneously and dynamically visualized with an animation. This critically facilitated the detection of patterns of gene expression changes that suggested underlying regulatory networks that a standard analysis by pairwise comparison and clustering could not have revealed.

**Conclusions:**

The results of the animation-assisted detection of changes in gene regulatory patterns make predictions about the potential roles of Hsp90 and its co-chaperone p23 in regulating whole sets of genes. The simultaneous dynamic visualization of microarray experiments, represented in networks built by integrating one's own experimental with publicly accessible data, represents a powerful discovery tool that allows the generation of new interpretations and hypotheses.

## Background

In the current post-genomic era, an increasing amount of data is generated by the application of high-throughput technologies. Expression profiling analyses using DNA microarray approaches are extensively used to study global changes in gene expression patterns of multiple cell types and tissues under different conditions. Moreover, there are publicly accessible databases containing the experimentally established DNA binding sequences for transcription factors (TF). To identify interaction partners of a protein of interest, several large-scale methods such as yeast two-hybrid screens, tandem affinity purification followed by mass spectrometric analyses, and protein chips are commonly used. Proteome-wide studies to identify protein-protein interaction (PPI) partners for many proteins have resulted in several publicly available and well-curated databases, which can be used to elucidate and to explore PPI networks. The integration of this large amount of data from many sources in a comprehensive and insightful way requires the use of computational tools to combine, to manipulate and to visualize the information [1-3]. Visualization constitutes in itself a challenge. This is particularly true for the multivariate type of data that omics approaches generate. A multitude of tools have been developed that allow both still and animated data visualization [4,5]. However, it is increasingly recognized that visualizing data is more than just presenting it; it also constitutes an exploration tool [6]. It could even be argued that the characteristics of human pattern recognition [7] make the human subject a powerful ally of mathematical algorithms for the discovery of principles in complex data.

Hsp90 is an abundant molecular chaperone that is essential for many cellular regulatory and signal transduction systems by promoting the functionally competent state of a large list of client proteins. Hsp90 recruits a cohort of associated partners or co-chaperones, which form a variety of multiprotein complexes with Hsp90. These co-chaperones assist Hsp90 by modulating its ATPase cycle and by facilitating its interactions with various client/substrate proteins [8-10]. An extensively studied Hsp90 co-chaperone is p23 (known as Sba1 in the budding yeast *Saccharomyces cerevisiae*) [11,12]. This ubiquitous acidic protein binds and stabilizes the ATP-bound dimeric form of Hsp90 [10,13,14], inhibits ATP hydrolysis and thereby traps Hsp90 in a state with high affinity for client proteins [15,16]. Hsp90 inhibitory drugs such as geldanamycin and radicicol, and post-translational modifications, such as the hyperacetylation of Hsp90, lead to the release of p23 from Hsp90, which destabilizes the Hsp90-substrate interaction [17,18]. Moreover, it was found that in the absence of p23, yeast and mammalian cells are viable [19,20], but become hypersensitive to Hsp90 inhibitors [21]. Considering the biochemically established important role of p23 in regulating Hsp90, we wanted to investigate the transcriptional adaptations of budding yeast when p23 is removed from the Hsp90 complex either genetically or pharmacologically. We carried out microarray experiments with wild-type and p23-deficient *S. cerevisiae *strains, in which Hsp90 function was additionally impaired with the Hsp90 inhibitor radicicol.

Early on, it became apparent that the classical pairwise comparison and subsequent clustering of the microarray data, based on changes in gene expression alone, would be insufficient to reveal more complex underlying regulatory patterns. We therefore linked and grouped the genes according to their relationships in expression across experiments, their interactions at the protein level, and their cellular localization. Moreover, we explored the possibility that some genes might be coregulated by the same TFs by searching for the presence of enriched TF binding sites in their promoters. All these approaches combined together allowed us to visualize and to detect regulatory patterns of genes affected (directly or indirectly) simultaneously by Hsp90 and p23. The results of these analyses are of heuristic value to build new hypotheses for further validation experiments.

## Methods

### RNA sample preparation and microarray analysis

The strain BY4741 (Mata *his3Δ1 leu2Δ0 met15Δ0 ura3Δ0) *was used as the wild-type strain and its derivative BYP2 [21] as the *Δsba1 *strain. The strains were grown in YEPD with 10 μM radicicol or with the same volume of the vehicle DMSO for about 14 hours to reach the desired OD_600 _≈ 0.6-1.0. RNA was extracted from triplicate cultures using the hot-phenol method and subsequently cleaned up further on columns with the QiaGen RNA purification Kit. RNA quality was assessed with a BioAnalyzer (Bio-Rad). Expression profiles for these four experimental conditions using triplicate samples were determined using the YG_S98 gene chips from Affymetrix at the genomics platform of the University of Geneva. The data are available from ArrayExpress at EBI (access code E-TABM-573). The raw data obtained from the expression profiling was analyzed with the software GeneSpring 7.3. The samples were subjected to a 2-way Anova statistical test and a cut-off of p-value < 0.05 with the volcano plot for pair-wise comparisons between two experimental conditions.

### TF binding site analysis

Sequence retrieval: 1000 nucleotides upstream of the transcription start sites were retrieved from the *S. cerevisiae *Refseq genomic DNA sequence available at NCBI for the set of genes characterized by a 1.5 fold change in expression when Hsp90 was inhibited with radicicol in wild-type cells (Additional File 1 Table S1). Similarly, 1000 nucleotides upstream of the transcription start sites for all Refseq annotated *S. cerevisiae *genes were retrieved to be used as the background/control sequences. TF identification: TRANSFAC Professional 11.4 was used as described previously [22] except that it was done with the set of all fungal TF binding sites retrieved from matrix.dat in TRANSFAC. The over-represented TF binding sites were sorted by their relative over-representation in the target sequence set (see Additional File 2 Table S2).

### Yeast reporter gene assays

The reporter gene assays were done with yeast strain YNK100 (relevant genotype: *pdr5-101*) [23] transformed with the plasmids pSTRE-LacZ(TRP1) [24] and pLG/Z [25], which contain the STRE from the *CTT1 *promoter and the UAS of the *GAL1 *promoter, respectively, upstream of a minimal *CYC1 *promoter driving β-galactosidase expression. The transformants with pSTRE-LacZ(TRP1) and pLG/Z were grown in YEP complemented with 2% glucose and 2% raffinose/2% glycerol, respectively. Overnight cultures were diluted to a density of OD_600 _= 0.3, incubated for 4 hours before adding 30 μM radicicol in ethanol or vehicle alone. In the case of the pLG/Z transformants, galactose was added at the same time to induce reporter gene expression. After an additional 3 hours of incubation, β-galactosidase activities were measured using standard protocols and normalized to cell densities (OD_600_). For all growth conditions and strains, the growth temperature was 25°C. The data points represent the averages of three and two independent experiments with replicates for the STRE and *GAL *reporters, respectively.

### Integration of microarray data with PPI networks

PPI network generation and expression data integration using Cytoscape (http://www.cytoscape.org) has been extensively described by Cline and colleagues [26]. Briefly, all the physical interactions annotated in the BioGrid database for *S. cerevisiae *(release 2.0.49) were formatted into an Excel spreadsheet and then uploaded into Cytoscape using the function "Import Network from a table". From this network, the first level of PPIs was retrieved for the list of genes showing significant fold change in all microarray experiments. One "node" in this network corresponds to a gene/protein and the connection between them is called "edge" and it refers to the interaction between these two nodes. The expression data was then loaded onto this network, importing the fold change values in a tab-delimited format. This allows one later to compute a correlation network using the Cytoscape plugin ExpressionCorrelation (http://www.baderlab.org/Software/ExpressionCorrelation). This plugin facilitates the assembly of a co-expression network, integrated into the PPI network, from microarray data, by computing the Pearson correlation coefficient for all pairwise comparisons. We used a cutoff of 0.9 in this analysis, and so, any correlations above these threshold values are displayed again as an edge between two nodes. Network images were generated using Cytoscape version 2.6.1 and each node was placed according to its annotated intracellular localization (Gene Ontology [GO] cellular component) in cellular compartment layers using the plugin Cerebral [27]. All the schemes were first exported as Cerebral views from Cytoscape and then loaded into Adobe Illustrator for editing. The animation was generated in Adobe Illustrator and exported as a Flash file.

## Results

### Microarray analysis and data organization in a graph

**Figure 1:**
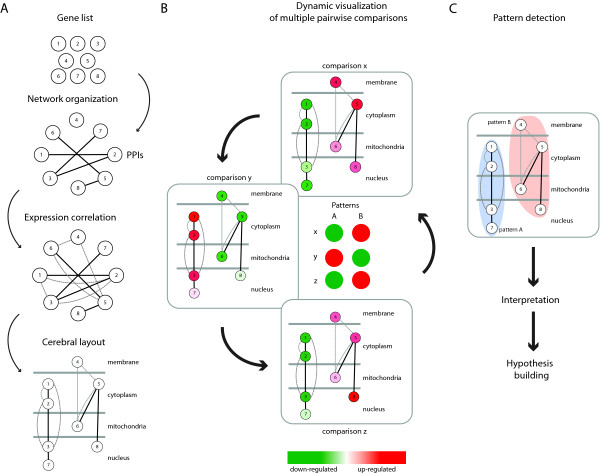
**Flow chart of the microarray data analysis**. To illustrate the procedure, we arbitrarily used a set of 8 genes, whose expression varies across 3 different pairwise experimental comparisons and whose corresponding proteins undergo a certain number of interactions. (A) Generating a set of networks based on pairwise comparisons of experimental conditions. The starting point for this procedure is an initial set of differentially regulated genes (Gene list). This gene list is used to generate a network where nodes are genes (or proteins), which are first connected by edges based on available protein-protein interaction information (PPIs, black lines). The network connectivity is further enriched by the addition of extra edges, which indicate that the expression of linked genes is correlated across all given experimental conditions (grey dashed lines). Thus, the latter is based on tracking expression correlation between genes in pairwise comparisons as shown in panel B. Finally, the layout of the network is organized using the Cytoscape plugin Cerebral. Here, nodes are organized based on their intracellular localization and their level of connectivity (PPI and expression correlation). (B) Dynamic visualization of network maps of pairwise comparisons. Nodes in the network maps are colored with a red-to-green gradient according to their expression values, along all the analyzed pairwise comparisons. Two different patterns (A and B) of gene expression behaviors emerge by moving between the different network panels (x, y, z). This can be greatly facilitated by generating a animation from these network panels. (C) Detected patterns can suggest biological interpretations and new hypothesis.

**Figure 2:**
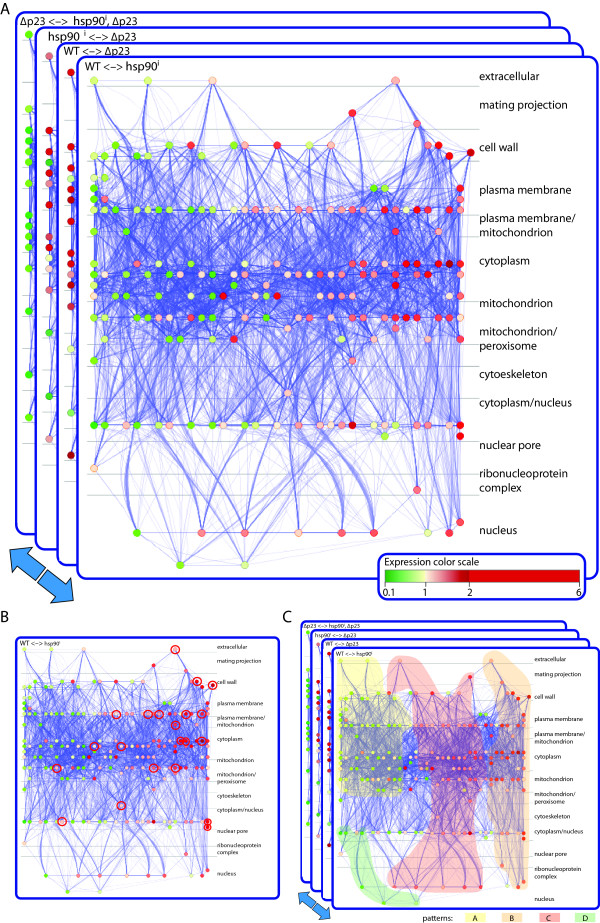
**Visualization of the pairwise comparisons of the microarray data by a dynamic graph**. Genes showing significant fold change in their expression levels in at least one pairwise comparison of all the experimental conditions were linked and grouped according to their coexpression levels along the experiments, their interactions at the protein levels (relatively few in this particular dataset) and their cellular localization. (A) Nodes represent genes (and gene products). These nodes are colored with a red-to-green gradient according to their expression/fold change values, where red and green represent up-regulation and down-regulation, respectively (see inset for color gradient). For dynamic visualization, these graphs were converted into an animation. (B) The TF enrichment analysis of genes that are up-regulated upon Hsp90 inhibition showed that most of the red nodes correspond to genes that are potentially regulated by STREs (highlighted red nodes). (C) The repeated observation of the dynamic graph (animation), allows the identification of groups of nodes sharing the same color patterns because of their particular expression levels across all experiments. The regions highlighted with pastel colors indicate the positions in the graph where a majority of the nodes defining each pattern were identified.

Using a microarray experiment, we evaluated gene expression responses in yeast when the function of Hsp90 was disrupted by radicicol (referred to as hsp90^**i **^in Tables and Figures) in the presence and/or absence of p23 (referred to as WT and Δp23, respectively). Note that radicicol has been widely used in budding yeast as a specific pharmacological inhibitor of Hsp90. Hsp90 is the only known target of radicicol *in vivo *in this organism, although it should not be ignored that it has been shown to bind a few other proteins with a related ATP binding fold *in vitro *or in other organisms [28-30]. Based on pairwise comparisons between different experimental conditions, a total of 185 genes (Additional File 1 Table S1) were found to be up- or down-regulated by 1.5 fold or more in at least one comparison. In order to make the information more intelligible, we organized the data, represented by the list of genes mentioned above, in networks based on additional information that could be obtained about these genes and their protein products from public databases. The flow chart of our new data analysis pipeline is presented in Figure 1. Genes are first organized graphically according to PPIs of their protein products based on data manually extracted from the BioGRID database [31]. Next, graph connectivity is further enriched by taking into account the pairwise differential changes in expression of the query genes for different conditions in the experiment. This information is superimposed on the PPI network (Figures 1A and 2). Thus, nodes represent genes (and gene products, *i.e.*, proteins), and edges represent physical interactions of the proteins or correlations of expression levels of the respective genes across all given conditions. Furthermore, the intracellular localization for each node (protein) is obtained from the Gene Ontology (GO) database [32] and this information is used to reorganize the graph further (Figures 1A and 2) using the layout provided by the Cytoscape plugin Cerebral [27]. With Cerebral, nodes get positioned according to their intracellular localization and their connectivity with topological neighbors; at the same time, they get "geographically" separated from unrelated nodes. As a result, highly interconnected nodes with similar intracellular localization get closer in the generated graph [27] (Figures 1A and 2). All these manipulations group the nodes in a graph according to their PPIs, co-expression, and intracellular localizations. Thereafter, nodes are colored with a red-to-green gradient according to their expression values, where red and green represented up-regulation and down-regulation, respectively (Figures 1B and 2A). At this point, individual dependency graphs [3] relate to a single pairwise comparison of experimental conditions and are more or less multivariate depending on what additional data were incorporated with the original gene expression profiles.

### Finding biologically relevant co-regulation patterns

**Figure 3:**
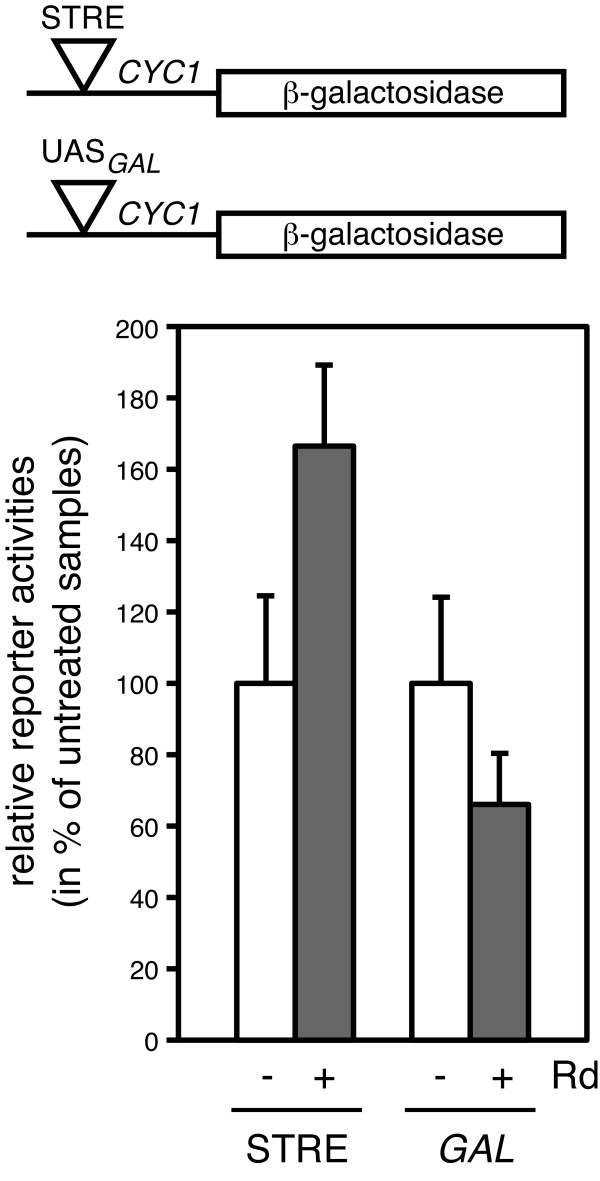
**Pharmacological inhibition of Hsp90 induces a stress response in yeast**. Expression of β-galactosidase from the stress-inducible STRE reporter and the galactose-inducible control plasmids (schematically shown at the top of the Figure) was assayed without (open columns) and with (grey columns) treatment with 30 μM radicicol (Rd). Activities are expressed as % of the activities of the respective untreated samples, arbitrarily set to 100%. The difference between treated and untreated samples in the case of the STRE reporter are statistically significant with a p-value of < 0.05.

To identify patterns of coregulation, graphs can be analyzed one experiment at a time (pairwise comparisons) or "dynamically" (all experiments together) (Figure 1B). Performing a GO analysis on the results of single pairwise comparisons of the expression data, notably the ones for wild-type cells treated or not with radicicol, we found that there are a significant number of genes related to the stress response (highlighted in Additional File 1 Table S1). A TF site analysis of the promoters of genes showing significant changes in expression upon inhibition of Hsp90 with radicicol revealed that they are considerably enriched in stress response elements (STREs) (Additional File 2 Table S2). Furthermore, the genes with STREs in their promoters (genes marked "yes" in the column STRE of Additional File 1 Table S1) all belong to the ones that are up-regulated upon pharmacological inhibition of Hsp90, indicating that they might be implicated in the same biological processes (Figure 2B, highlighted red nodes, and Additional File 1 Table S1). The enrichment of STREs in promoters of many genes up-regulated by Hsp90 inhibition suggested that inhibition of Hsp90 may lead to a stress response by signaling through these regulatory elements. To evaluate this hypothesis experimentally, we examined the expression of a STRE reporter gene following Hsp90 inhibition by radicicol and found that inhibition of Hsp90 indeed up-regulates it (Figure 3). These results support this hypothesis, which was based on a standard pairwise comparison and TF analysis.

Whereas the more or less multidimensional dependency graphs generated by these binary comparisons are useful and yield interesting results, some patterns might only become apparent by investigating the data of all performed experiments simultaneously as highlighted in Figure 1. Specifically, looking at all experiments at once may facilitate the detection of genes or sets of genes with similar or particular expression patterns across the different genetic and pharmacological conditions (Figure 1B). To this end, all the networks generated from the different pairwise comparisons were superimposed on each other as a stack and converted into a dynamic graph or animation (Additional File 3 Movie S1). This animation then served as a discovery tool to extract novel patterns of behavior. By visualizing the recurring patterns of colors (red or green according to their expression values), genes could be assigned to different clusters (Figure 1B), which are coordinately or simultaneously regulated by Hsp90 and p23 (Figure 2C and Additional File 3 Movie S1). In Figure 2C and Additional File 3 Movie S1, highlighted regions indicate the positions in the graph where most of the nodes belonging to each pattern were visually identified. For example, by watching the animation (Additional File 3 Movie S1), one can observe the following pattern of node color changes in the central/upper left region of the graph: red, green, green and red (arbitrarily referred to as pattern A). Similarly, there is another pattern in the central/upper right region: green, red, red and green (pattern B). The classification of all genes in our study according to these patterns is given in column "Pattern" of the Additional File 1 Table S1.

**Figure 4:**
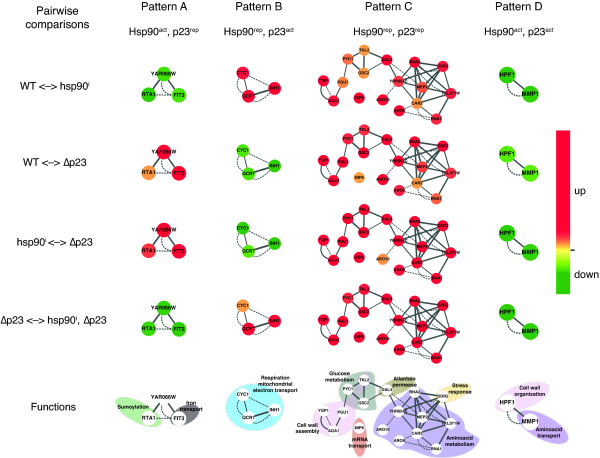
**Genes that change significantly in all pairwise comparisons define the four regulatory patterns**. Genes whose expression changed significantly in all pairwise comparisons (Additional File 4 Table S3) are organized in a graph according to their level of coexpression (grey lines connecting nodes; edge width is representative of the extent of coexpression) and similar cellular localization of the respective proteins (grey dashed lines). Note that for this particular set of genes, there are no known PPIs. The changes in the color-coded expression values of these genes (see color gradient on the right) across the four pairwise comparisons define the patterns A to D. The terms Hsp90^act^, Hsp90^rep^, p23^act ^and p23^rep ^refer to the hypothesized gene regulatory activities of Hsp90 and p23 as direct or indirect activators or repressors. Some of the cellular functions (GO terms) for the same set of genes are indicated at the bottom.

Thus, it is the visual impression of animated color changes of clusters of nodes, which allows one to identify potential regulatory patterns. The order of the color changes for a given node is determined by the order of the graphs used to generate the animation. Whereas the order is irrelevant for our particular data set, we do not exclude that it may be important in others (for example, in time course experiments). Amongst all of the genes represented in the full data set, it is of course particularly those whose expression changes substantially (indicated by the red and green nodes) across all pairwise comparisons that define patterns (see Figure 4 and Additional File 4 Table S3). However, considering that this is a relatively small subset of genes, notably in our data set, it is important to realize that the detection of patterns is crucially aided by many more genes (nodes) that change substantially in only a subset of pairwise comparisons (and less or not at all in others).

**Figure 5:**
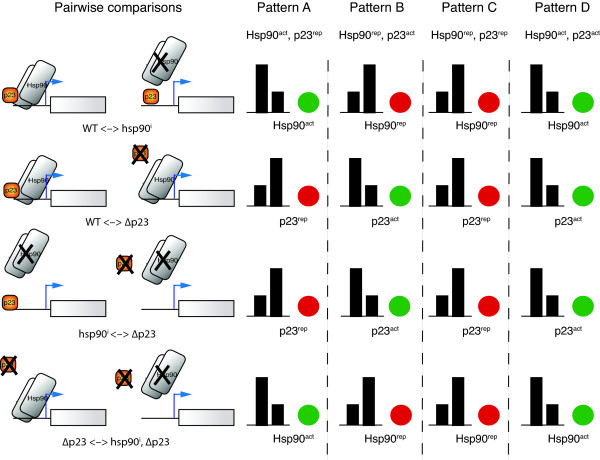
**Biological interpretation of color patterns identified in the animation-assisted network analysis**. The left side shows the biological interpretation of the assayed conditions, where Hsp90 was inhibited (or not) in wild-type or p23 -deficient yeast strains. Genes showing significant fold change under these conditions are genes affected (directly or indirectly, positively or negatively) by Hsp90 and p23. On the right, the bar graphs illustrate the expected expression levels of any given gene in each condition of the pairwise comparisons, according to the observed final fold change (red or green colors). In the animation-assisted analysis (visualizing all four pairwise comparisons in a animation), there are genes which follow a defined behavior along all four comparisons, that is these genes display a defined pattern of red/green colors (according to their expression values). If a gene in the first two comparisons is first green and then red it means that the inhibition of Hsp90 leads to a decrease in its expression levels but the disruption of p23 results in an increase, which would be in accordance with a gene positively regulated by Hsp90 and negatively by p23. A similar reasoning can be applied to all other patterns.

The original biological set-up and rationale of the microarray experiments then serve to interpret these novel patterns (Figures 1C and 5). For example, we can deduce that pattern A corresponds to a set of genes simultaneously affected (directly or indirectly) by Hsp90 and p23, positively by Hsp90 and negatively by p23 (referred to as Hsp90^act ^and p23^rep^, respectively, in Figures 4 and 5). Doing the same analysis for pattern B, it could be inferred that these genes would be affected negatively by Hsp90 and positively by p23 (Figure 5). Furthermore, there is a pattern (pattern C) in which genes are up-regulated in all experiments: all nodes are red in all comparisons (central region of the graph, Figure 2C and Additional File 3 Movie S1) implying that both Hsp90 and p23 negatively affect genes in this pattern. Pattern D is defined by genes that are down-regulated in all experiments: all nodes are green in all comparisons (left lower region), indicating that all the genes in this pattern might be positively affected by Hsp90 and p23 (Figure 5). Not unexpectedly, there were several genes (group E) that did not have any defined pattern of expression among all the pairwise comparisons (Additional File 1 Table S1). Although Hsp90 and p23 regulate these genes, no clear insight emerges with this type of analysis for their concurrent/simultaneous regulation by both of these proteins.

### Inference of functions for patterns differentially regulated by Hsp90 and p23

Following the identification of different patterns by this animation, we extracted the genes corresponding to each pattern (Additional File 1 Table S1). Full GO data for genes belonging to each pattern were retrieved to address the potential functions of these groups. Genes from pattern A (Hsp90^act^, p23^rep^) showed enrichment in functional terms associated with iron and ion homeostasis, whereas functions of those belonging to pattern B (Hsp90^rep^, p23^act^) fell into a mitochondrial function (respiration), kinase activity dependent on cyclins and progression through the cell cycle. Genes from pattern C (Hsp90^rep^, p23^rep^) exhibit GO terms related with conjugation processes, catabolism of nitrogen sources and response to stress. The concomitant absence of Hsp90 and p23 induces the expression of stress-related proteins like Ssa4 (encoding one of the cytosolic Hsp70 isoforms), Hsp26, Hsp31, Ddr2. Interestingly, both chaperones might have a positive influence on the expression of genes (pattern D) related with mitochondrial protein import and carbon source metabolism (*TOM70*, *OAC1*, *MDH2 *and *DIC1*), a phosphatase that positively regulates the Hsp90 chaperone machinery (Ppt1) [33], or chromatin remodelers like Nhp6 and Imd2. Furthermore, the genes lacking any particular pattern (group E), do not show significant enrichment for any functional annotations. Interestingly, the genes for Hsp90 (*HSP82 *encoding one of the two cytosolic isoforms) and for its co-chaperones Aha1, Cpr6 and Sti1 belong to this group of genes, thus implying that their regulation is not affected by the interplay between Hsp90 and p23.

## Discussion

Here we have presented a novel approach to integrate experimental gene expression profiles with already available data. We were able to combine our own data from microarray experiments and data from public databases of PPIs, TF, and GO in a rational manner to address the global biological functions of Hsp90 and p23 both individually and, perhaps more interestingly, concomitantly.

Several key features of our working pipeline (Figure 1) allowed us to process and to understand certain aspects of our data. First, we worked with genes showing a significant fold change in at least one, often several pairwise comparisons that were analyzed (Figure 1A). Second, we organized them into a graph establishing connections between nodes according to their co-expression levels and their ability to interact at the protein level (Figure 1A). Third, we organized the nodes in the graph according to their annotated intracellular localization. Using the Cerebral layout, highly interconnected nodes with the same intracellular localization get grouped in the final individual graphs [27] (Figure 1A). And finally, a novel and essential feature of the analysis was to convert all the generated graphs into an animation to facilitate the visualization of similar expression patterns of genes topologically grouped in the individual graphs (Figures 1B and 2A, and Additional File 3 Movie S1). It is important to emphasize that this pattern detection was only possible by this method and that we failed to detect these patterns with more standard clustering algorithms (data not shown). The latter may have discarded or "undervalued" nodes with only marginal changes in gene expression whereas a human subject's pattern recognition would have incorporated some of these as well. While small fold changes may not have any significant value by themselves, as part of a larger animated pattern, they attract the attention of a human observer and they may become corroborative. The interpretation of these patterns matched the information contained in the pairwise comparisons of the microarray data (Figure 5), which finally allowed us to uncover the genes that are regulated by both Hsp90 and p23. Thus, the use of an animation to visualize patterns was a key tool to connect the data of the different microarray experiments.

Once the networks had been built, we were able to perform two kinds of analyses, single pairwise ones and an animation-assisted one that takes advantage of all pairwise comparisons. With the single analysis we found that 114 genes were up-regulated upon pharmacological Hsp90 inhibition (Figure 2B, red nodes and Additional File 1 Table S1, genes boxed in blue). Of these, 26 presented an enrichment of GO terms related with stress response (Additional File 1 Table S1, genes highlighted in orange), in accordance with the finding that these genes contained an increased occurrence of STREs in their promoters. The stress response in yeast is regulated by the TFs Hsf1 and Msn2/Msn4, which bind to promoters containing heat-shock response elements and STREs, respectively [34,35]. Some of these promoters could also contain both elements [36,37]. From our study, it appears that the sole inhibition of Hsp90 is sufficient to up-regulate stress-related genes with STREs in their promoters. This would indicate that Hsp90 influences the Msn2/Msn4 system. Analyzing the yeast PPI networks extracted from public repositories and the literature, we can infer that Hsp90 (and co-chaperones) interacts with kinases known to repress the Msn2/Msn4 system (and the stress response) [38-41] (Additional File 5 Table S4). Since many kinases are known Hsp90 clients (see regularly updated list at http://www.picard.ch/downloads/downloads.htm), we can speculate that Hsp90 and its co-chaperones maintain kinases responsible for Msn2/Msn4 repression in a functional state thereby regulating the stress-response (Additional File 6 Figure S1). Interestingly, with animation-assisted analysis, we noticed that not all stress-related genes belong to the same expression pattern. Most of these genes belong to patterns B and C, indicating that they are at the same time affected by Hsp90 and p23 (Additional File 3 Movie S1). It is also clear that Hsp90 and other co-chaperones (Sti1, Aha1, Cpr6) are up-regulated upon pharmacological Hsp90 inhibition, and yet they do not show any particular pattern. Hsf1 is the only stress-inducible transcriptional regulator of Hsp90 protein expression in yeast [42,43]. Conversely, Hsp90 represses gene expression from Hsf1-dependent promoters [44], establishing a negative feedback loop for its own expression. Conceivably, the genes for Hsp90 and at least some of its co-chaperones are regulated only by Hsf1, while the other stress-related genes exhibit a more complex regulation involving the concurrent action of Hsp90 and p23, potentially through the Msn2/Msn4 system.

## Conclusions

The animation-assisted analysis involving the visualization of complex connection maps with an animation uniquely allowed us to integrate the information from all the pairwise comparisons that were possible with the microarray data obtained in this study. We were able to address the potential actions of Hsp90 and p23 for the expression of a large list of genes. Moreover, we could gain insights about groups of genes that are regulated together or individually by these two proteins. Even though Hsp90 and p23 seem to be essential partners for most of their functions, it is becoming clear that they can exert both coordinated as well as opposite influences on some of their clients, as previously described for example for the regulation of the telomerase complex or for recycling of nuclear receptors at chromatin targets [[45,46], and reviewed in ref. [47]]. Independent or even opposite influences are suggested by gene expression patterns A and B. It is conceivable, for example, that Hsp90, in association with other co-chaperones, is required to assist a particular transcription factor or complex at a set of target genes while the primary role of p23 for the same set of genes might be to disrupt transcription factor complexes. Taking into account indirect pathways, many more schemes are possible as well. At this point, we can only speculate about the underlying mechanisms. It will be both challenging and interesting to dissect them experimentally both in yeast and in other organisms.

Our new approach might be appropriate to process a variety of microarray data. Following enrichment with biological annotations from several other sources such as PPI, TF, GO and localization databases, the dynamic visualization represents an additional powerful discovery tool. It remains to be tested to what extent it can be more generally applied, notably to even larger sets of genes or different experimental conditions. Although it may ultimately be possible to replace the reiterated visual inspection of the animation with an algorithm that would automatically identify patterns in multiple dependency graphs, we contend that the human eye and brain are currently simpler clustering tools that are also more accessible to a wide range of wet bench scientists.

## Funding

This work was supported by the Canton de Genève, the Swiss National Science Foundation, and the Fondation Medic.

## Authors' contributions

Conceived and designed the project: PCE FF DP. Performed the wet bench experiments: FF GM. Performed the bioinformatic analyses: PCE FF DPP DP. Wrote the paper: PCE DP. All authors have read and approved the final manuscript.

## Competing interests

The authors declare that they have no competing interests.

## Supplementary Material

Additional file 1 **List of genes showing a significant fold change in the microarray data in at least one pairwise comparison**. This file contains all the relevant microarray data. Click here for file

Additional file 2 **Overrepresented TF-binding sites in promoters of genes showing significant changes in expression upon Hsp90 inhibition**. Click here for file

Additional file 3 **Animation allowing a dynamic analysis of the four pairwise comparisons**. This movie can most easily be viewed by opening it in a web browser. Click here for file

Additional file 4 **List of genes showing a significant fold change in all pairwise comparisons**. This file contains a subset of the data of Additional File 1 Table S1. Click here for file

Additional file 5 **Interactions between the Msn2/Msn4 and Hsp90-Hsp70 molecular chaperone systems**. This file contains a list of known PPIs and a graphical representation of them. Click here for file

Additional file 6 **Speculative model of the regulation of the Msn2/Msn4 system by Hsp90 and p23**. Black lines (--) illustrate PPIs retrieved from public databases (with experimentally determined interactions; see Additional File 5 Table S4 for details). Arrows and blunt arrows indicate activation and repression, respectively. Click here for file
